# Facile purification of colloidal NIR-responsive gold nanorods using ions assisted self-assembly

**DOI:** 10.1186/1556-276X-6-143

**Published:** 2011-02-14

**Authors:** Lianke Liu, Zhirui Guo, Lina Xu, Ruizhi Xu, Xiang Lu

**Affiliations:** 1 Department of Oncology, the First Affiliated Hospital of Nanjing Medical University, Nanjing 210029, China; 2 The Second Affiliated Hospital of Nanjing Medical University, Nanjing 210011, China; 3 State Key Laboratory of Bioelectronics, Southeast University, Nanjing 210096, China; 4 Department of Radiotherapy, the First Affiliated Hospital of Nanjing Medical University, Nanjing 210029, China

## Abstract

Anisotropic metal nanoparticles have been paid much attention because the broken symmetry of these nanoparticles often leads to novel properties. Anisotropic gold nanoparticles obtained by wet chemical methods inevitably accompany spherical ones due to the intrinsically high symmetry of face-centred cubic metal. Therefore, it is essential for the purification of anisotropic gold nanoparticles. This work presents a facile, low cost while effective solution to the challenging issue of high-purity separation of seed-mediated grown NIR-responsive gold nanorods from co-produced spherical and cubic nanoparticles in solution. The key point of our strategy lies in different shape-dependent solution stability between anisotropic nanoparticles and symmetric ones and selective self-assembly and subsequent precipitation can be induced by introducing ions to the as-made nanorod solution. As a result, gold nanorods of excellent purity (97% in number density) have been obtained within a short time, which has been confirmed by SEM observation and UV-vis-NIR spectroscopy respectively. Based on the experimental facts, a possible shape separation mechanism was also proposed.

## Introduction

Metal nanoparticles with specific shape have become the focus of intensive research because the physicochemical properties of these nanoparticles are highly dependent on their shapes and exposed crystal facets [1,2]. Of the possible shapes of metal nanoparticles, rod-shaped gold nanoparticles are especially attractive as they offer unique optical properties together with excellent adjustability and biocompatibility [3]. For gold nanorods (NRs), their plasmon band corresponds to absorption and scattering of light is split in two due to their anisotropic shape: one weak band at higher energy resonates along the transverse axis of the NR, while the other strong band at lower energy resonates along the longitudinal axis of the NR. The transverse band is located in the visible region of the electromagnetic spectrum at ca. 510 nm and is insensitive to the change of the aspect ratio (length divided by width) of the NRs, while the longitudinal band can be drastically tailored from the visible to the near-infrared (NIR) region (700-1,100 nm) by increasing the aspect ratios. Due to the high transmission of the NIR lights in tissue, blood, and water, colloidal NIR-responsive gold NRs have been widely explored for biomedical imaging and photothermal therapy of tumors [4-7]. On the other hand, the longitudinal plasmon band of gold NRs shows excellent sensitivity to the changes of the local dielectric surroundings, including solvent, adsorbed molecules and the aggregate state and the sensitivity is found to be largely improved when increasing the aspect ratio, enabling gold NRs for versatile sensing applications [8-10]. Furthermore, since the transverse plasmon band of gold NRs is basically immune of the change of aspect ratios, one can use a dispersion with a composite longitudinal plasmon bands by mixing gold NRs with proper aspect ratios to perform multiplex sensing in solution [11,12].

Colloidal gold NRs have been synthesized by a variety of methods such as templating [13], electrochemistry [14], photochemistry [15] and seeding [16]. The seed-mediated growth procedure in the presence of surfactant has been most popular due to no need of specialized equipment or organic solvents, high yield of NRs, and convenient particle aspect ratio control. The current routine procedure is originated in 2001 by Jana et al [17] and further improved in 2003 by Nikoobakht et al [18]. Briefly, in a single-surfactant system, approximately 4 nm gold spherical nanoparticles are used as the seeds and subsequent reduction of HAuCl_4 _with ascorbic acid in the presence of a cationic surfactant cetyltrimethylammonium bromide (CTAB) as growth solution. A small amount of AgNO_3 _is put into the growth solution before seed addition to direct the rod growth and adjust the aspect ratio. This procedure results in reproducible formation of gold NRs with aspect ratios from 1.5 to 4.5 in nearly quantitative yields (approximately 99%) and their longitudinal plasmon bands of these NRs are mainly located in visible region. To obtain gold NRs with higher aspect ratios, a co-surfactant of benzyldimethylhexadecylammonium chloride (BDAC) has to be introduced in the growth solution. The CTAB/BDAC system produces NRs with aspect ratios ranging from 5 up to 10 and their longitudinal plasmon bands are located in NIR region. Unfortunately, this binary surfactant system also co-produce quite a few of symmetric gold nanoparticles including spherical nanoparticles (strictly, truncated octahedral nanocrystals) and cubic nanoparticles as byproducts. In order to take full advantage of the potentials offered by the NIR-responsive gold NRs, high-purity separation is necessary before employing them. Unlike longer gold NRs with aspect ratios beyond 10 which undergo gravitational settling from solution [19], colloidal gold NRs do not precipitate spontaneously because their gravitational force is insignificant as compared to Brownian motion. To solve this long-existing problem, Sharma et al adopted centrifugation-assisted sedimentation to purify these NIR-responsive gold NRs by the different shape-dependent sedimentation coefficient of the nanoparticles [20]. However, this technology is limited to the applicability of very low concentration of nanoparticle mixtures. Very recently, Park et al demonstrated an efficient procedure for separating gold NRs from spherical or cubic nanoparticles through the formation of NR flocculates by surfactant micelle-induced depletion interaction [21]. In their procedure, a large quantity of CTAB or CTAB/BDAC mixture was demanded to add into the as-made NRs solution to form supersaturated micelles to lead to phase separation between the NRs and the symmetric ones. Herein, we present a low-cost, non-toxic while facile method where colloidal gold NRs can be separated from the co-produced symmetric nanoparticles through partially electrostatic shielding by the addition of proper amount of NaCl at ambient conditions. It has found that symmetric nanoparticles hold much better solution stability under a higher ionic concentration and still remain in the solution, while NRs subject to self-assembly in a side-by-side mode and subsequently precipitation. This strategy allows for scale up separation of NRs that were present in the crude solution within a short time and leads to an excellent purity level at no less than 97%.

## Experimental section

Doubly distilled deionized water was used in all experiments. Cetyltrimethylammonium bromide (CTAB, 99%, Cat No: H6269) was from Sigma and benzyldimethylhexadecylammonium chloride (BDAC, >95%, Cat No: B0237) was from TCI. HAuCl_4_·4H_2_O, AgNO_3_, NaBH_4_, L-ascorbic acid and NaCl were all purchased from Shanghai Sinopharm Chemical Reagent Co. Ltd (China). All the glassware was cleaned by aqua regia (HCl:HNO_3 _in a 3:1 ratio by volume) and rinsed with water prior to the experiments.

Gold seeds were synthesized by adding 0.6 mL of ice-cold 10 mM NaBH_4 _to 10 mL of 0.25 mM HAuCl_4 _prepared in 0.1 M CTAB solution, following vigorous stirring for 2 min. The yellow colour changed immediately to brown, indicating the formation of small gold nanoparticles. The seed solution was aged for at least 1 h to ensure the complete hydrolysis of unreacted NaBH_4 _before further usage. The growth solution was prepared by mixing HAuCl_4 _(2 mL, 0.025 M), AgNO_3 _(1 mL, 0.01 M), CTAB (50 mL, 0.2 M), BDAC (50 ml, 0.2 M) at room temperature. Next, ascorbic acid (0.55 ml, 0.1 M) was added to the growth solution as a mild reducing agent, following the addition of seed solution (0.12 mL). The colour of the growth solution slowly changed from clear to red indicating the generation of gold nanoparticles.

For a typical separation procedure of gold NRs, a 20 mL of the as-made gold NR solution was firstly centrifuged at 16,500 × *g*/min for 20 min at room temperature in order to get rid of the extra CTAB and BDAC molecules. This operation was indispensable because that BDAC solution, while in high concentration (0.1 M), subjects to high viscosity upon adding salt solution and thus inhibits the separation of NRs. After centrifugation, the precipitates contained gold NRs and by-products were dispersed by deionized water to reach a volume of 20 mL again. The total concentration of the residual CTAB and BDAC in solution is approximately 5 mM. To this solution was added by a 20 mL of 1.72 M NaCl aqueous solution. The mixture was then kept for 4 h at ambient temperature without disturbance. Most of gold NRs deposited on the bottom of the beaker during the age time, which can be collected by carefully pouring out the supernatant. These precipitates were re-dispersed to form colloidal dispersion by a brief ultrasonication for further characterization. In order to gain an insight into the aggregation modes of the NRs in the as-collected precipitates, additional experiments have been done to acquire images of the intact precipitates by placing small pieces of silicon wafer on the bottom of the gold NR mixture solution before introducing NaCl solution. After the separation procedure, these silicon wafers were dried at ambient condition.

The gold samples were characterized by a Carl Zeiss Ultra Plus Field Emission Scanning Electron Microscope (Carl Zeiss NTS GmbH, Oberkochen, Germany) with an accelerating voltage of 20.0 kV. The UV-vis-NIR absorption spectra of the gold nanoparticle solutions were recorded by a UNICO 2802S spectrophotometer (UNICO, Shanghai, China) in a wavelength range of 300 to 1,100 nm.

## Results and discussion

**Figure 1:**
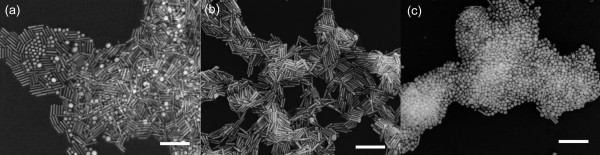
**SEM images of gold nanoparticles obtained by seed-mediated method**. **(a) **as-made gold NR mixture; **(b) **purified gold NRs from the mixture shown in (a); **(c) **nanoparticles kept in supernatant after purification. All scale bars are 200 nm

Typical SEM images of as-made gold NRs before and after purification are given in Figure 1. The NRs have an average diameter of 10 nm and the average aspect ratio of 6.5, while the impurities are mainly quasi-spherical nanparticles with diameters ranging from 15 to 30 nm and cubic nanoparticles with an edge length of approximately 20 nm (Figure 1a). After purification, the content of impurities in NRs was found to be less than 3% in number density (Figure 1b). Furthermore, the number fraction of NRs decreased from approximately 78% for the as-made nanoparticles to approximately 15% for the nanoparticles kept in supernatant after purification (Figure 1c), suggesting that more than 80% of as-made gold NRs have been recovered through a single circle of the purification procedure.

**Figure 2:**
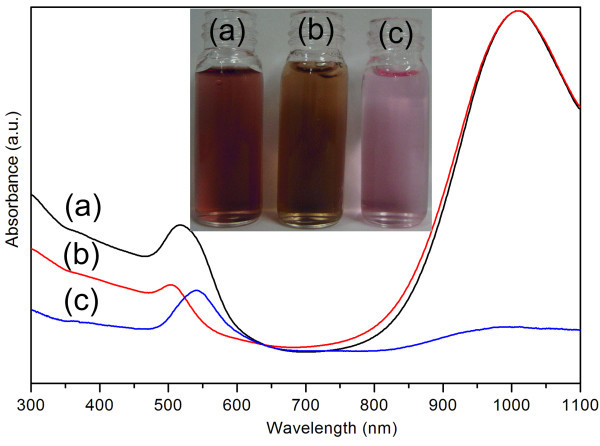
**Absorption spectra of aqueous solutions**. **(a) **As-made gold NR mixture, **(b) **purified gold NRs (red curve), and (c) nanoparticles kept in supernatant after purification. The photograph (inset) shows the corresponding colour of NR mixture solution, purified NRs solution and supernatant from left to right

To further investigate the purity of the separated gold NRs and better assess the efficiency of the present purification strategy, the as-made NR solution, the solution of the NR precipitates and the supernatant after purification were characterized by UV-vis-NIR spectroscopy respectively. For easy comparison, longitudinal plamson band of the purified NRs was normalized to the same intensity of that of the as-made NRs. As shown in Figure 2, the longitudinal plasmon bands of the NRs before and after purification locate at the similar position at 1,015 nm (Figure 2, curve a and b), whereas the transverse plasmon band of the purified NRs turns much narrower and blue shifts from 520 nm to 505 nm. We also observed that the transverse plasmon band of purified NRs turns much weaker than that of as-made NRs, indicating the exclusion of the symmetric nanoparticles. Correspondingly, the colour of the NR solutions also change from red to brown before and after purification due to high or minimal fraction of symmetric nanoparticles (Figure 2, inset). UV-vis-NIR spectrum of the supernatant also shows two plasmon bands: one sharp band at 540 nm and the other broad but weak band at 985 nm, respectively (Figure 2, curve c). The former band can be attributed to the symmetric nanoparticles remained in the supernatant. Since the two plasmon bands are independent of each other, it can therefore be concluded that the latter band is due to the longitudinal plasmon of NRs but not the linear assembly of the symmetric ones [22,23]. Moreover, the very weak intensity of the latter band also clearly reveals that the amount of NRs kept in supernatant is minimal, consistent with the SEM observation (Figure 1c). The above experimental facts confirm that the separated gold NRs are in high purity and the good efficiency of this purification strategy.

**Figure 3:**
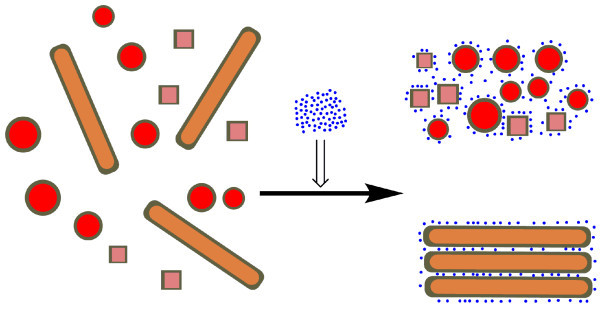
**A schematic illustration for ions-assisted selective separation of gold NRs**. The blue dots represent anions and other cartoons correspond to the positively charged bilayer of surfactants stabilized gold nanoparticles with different shapes

**Figure 4:**
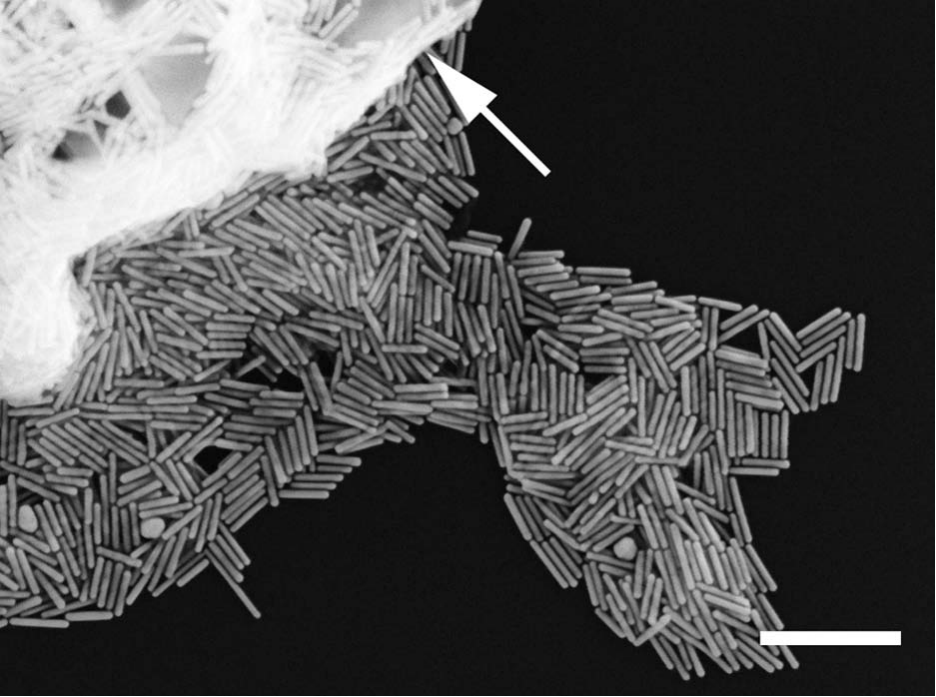
**SEM image of intact gold NR precipitates on a silicon wafer**. The large white domain shown by arrow is due to salt crystals formed during the drying process. Scale bar is 200 nm

The present strategy for selective separation of gold NRs is believed to largely benefit from significantly different shape-dependent solution stability between anisotropic gold nanparticles and symmetric ones under a higher ion concentration. According to the synthetic strategy we adopted, these as-made gold nanoparticles are protected by the positive-charged bilayer along the gold surface, which attribute to the cationic surfactant CTAB and BDAC [24]. Based on the classic Derjaguin-Landau-Vervey-Overbeek (DLVO) theory, the aqueous solution stability of colloidal particles depends on the interaction of the electrostatic repulsive potential (*V*_elec_) and the van der Waals attractive potential (*V*_vdW_) [25]. Upon adding proper amount of NaCl to the nanoparticle solution, the positively charged gold surfaces are partially shielded by Cl^-^, which induce the decrease of the electrostatic repulsion as well as the thickness of the electrical double layer of nanoparticles. Therefore, the *V*_vdW _might dominate the *V*_elec _and the nanoparticles could reach much shorter distance in which aggregation becomes more possible. Previous high-resolution TEM studies have verified that the shape of gold NRs are elongated polyhedrons enclosed by {100} and {110} facets on the sides and (001) and {111} facets at the ends [26,27]. In contrast to the symmetric nanoparticles with similar diameter (or width) having a minimal contact with convex nanoparticles, the NRs offer a much larger lateral surface area for contacting each other in a side-by-side mode. Based on the discussion above, we reasonably speculate that gold NRs hold higher aggregation potential than those of the co-produced symmetric nanoparticles if the distance between nanoparticles was shortened. As a result, the oriented aggregation and subsequent precipitation of NRs were induced by a higher ion concentration while keeping most of the symmetric nanoparticles in solution (illustrated in Figure 3). Moreover, the apparently blue shift of the longitudinal band of NRs contained in supernatant (Figure 2, curve 3) also suggested the side-by-side linkage of these NRs in solution [28]. Also noteworthy is that SEM observation on the intact NR precipitates (see experimental section) indicated the preferential side-by-side assembly mode (Figure 4).

**Figure 5:**
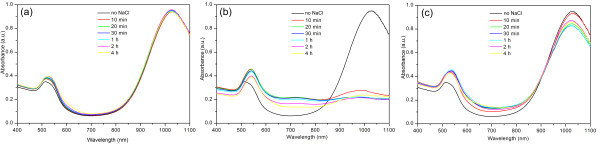
**Time resolved absorption spectra of as-made gold NR mixtures**. In the presence of NaCl with a certain concentration of (a) 0.43 M (b) 0.86 M and (c) 2.58 M, respectively. In each panel, the spectrum of NR mixtures without the presence of NaCl was also added for ease of comparison

To investigate the details of the ion-induced separation process of gold NRs, we studied the kinetic separation processes and the ion concentration correlation at different time stages. The separation procedures in the presence of different ion concentrations were similar with previous description (see experimental section), except that the concentration of NaCl solution was changed. As shown in Figure 5a, the two plasmon bands of the NRs mixture changed slightly during the whole age time under 0.43 M NaCl, indicating that most of these nanoparticle mixtures still keep stable in solution under present ion concentration. When increasing the concentration of NaCl to 0.86 M, the intensity of the longitudinal band of NRs at approximately 1,015 nm dramatically dropped within 10 min and then slowly decreased while the intensity of the plasmon band at initial 520 nm underwent an apparent increase during the initial 10 min and then slightly decreased (Figure 5b). These results indicate that selective precipitation of NRs occurred under the present ion concentration while keeping most of the symmetric ones in the solution, which is also consistent with SEM observation (Figure 1). However, when further increasing the concentration of NaCl as high as 2.58 M, the two plasmon bands only had a minimal decrease of the intensity during the entire age time, indicating that both NRs and the co-produced symmetric nanoparticles still keep their solution ability. Similarly, Sethi et al also found that minimal to no aggregation of the short gold NRs in solution was observed at higher buffer concentrations [29]. This phenomenon can be explained as follows: At a much higher salt concentration, the anions are sufficient enough for complete binding the positive charged surface of individual nanoparticle, leading to neutralization of the surface charges. As a result, an electronic double layer would form along the particle surface and re-introduces a repulsive force between the nanoparticles and prevents aggregation. Considering this regard, it is necessary for choosing the suitable window of the salt concentration to realize selective separation.

## Conclusions

In summary, we have found a new strategy to successfully separate colloidal NIR-responsive gold NRs from the co-produced symmetric nanoparticles to achieve a purity level of 97%. This purification strategy lies in the different shape-dependent solution stability between anisotropic nanoparticles and symmetric nanoparticles under a higher ion concentration. By post-adding salt solution, the charged gold surfaces are partially electrostatic shielded and thus the distance between nanoparticles was greatly shortened in which aggregation turns more favourable. As a result, preferential assembly and subsequent precipitation of NRs occurred spontaneously due to their large interrod contact area while keeping most of the symmetric nanoparticles in solution. These colloidal NIR-responsive gold NRs with high purity would further favour their usage in biomedical or nanotechnological fields. Since this purification strategy is efficient, scalable while non-destructive, we hope it could also be extended to the purification of other anisotropic nanoparticle systems not limited to gold.

## Competing interests

The authors declare that they have no competing interests.

## Authors' contributions

LL and ZG synthesized the gold nanorods, carried out the separation and spectra recording experiments and did data analysis. LX did SEM observation and data analysis. RX helped with the analysis of the mechanism for shape separation. XL conceived of the study and prepared different versions of the manuscript. All authors read and approved the final manuscript.
